# Comparison of Bolton’s Ratios before and after Treatment in an Iranian Population

**DOI:** 10.5681/joddd.2013.005

**Published:** 2013-02-21

**Authors:** Asghar Ebadifar, Rojin Taliee

**Affiliations:** ^1^Dentofacial Deformities Research Center Dental School, Shahid Beheshti University of Medical Sciences, Tehran, Iran; ^2^Assistant Professor, Deptartment of Orthodontics, Dental School, Shahid Beheshti University of Medical Sciences, Tehran, Iran; ^3^Postgraduate Student, Department of Periodontics, Dental School, Shahid Beheshti University of Medical Sciences, Tehran, Iran

**Keywords:** Anterior Bolton Index, mesiodistal width of tooth, orthodontic treatment planning, Total Bolton Index

## Abstract

**Background and aims:**

The correct relationship of the total mesiodistal width of the mandibular teeth to that of the maxillary teeth (Bolton’s ratios) is among the main factors considered in obtaining an optimal occlusion. The present study was conducted to determining the Bolton’s ratios before and after treatment in skeletal class I patients.

**Materials and methods:**

In this descriptive analytical study, 200 study casts of 100 patients (including 73 females and 27 males) were selected from 1,700 patient files with fixed, non-extraction orthodontic treatment protocol. The greatest mesiodistal width of teeth was measured using a digital caliper. The total Bolton index (TBI) and anterior Bolton index (ABI) were calculated for subjects and the obtained results were compared with the values reported by Bolton using one-sample t-test. The alterations in these indexes before and after treatment were compared with paired t-test.

**Results:**

Post-treatment ABI and TBI in patients were 77.35% ± 4.12% and 91.2% ± 1.66%, respectively. No significant difference was detected in ABI and TBI of patients after treatment when compared with the results of Bolton’s study (P > 0.05). However, a significant correlation was observed in values of ABI (P < 0.001) and TBI (P < 0.01) before and after treatment.

**Conclusion:**

These findings suggest that patients with mild hypodontia have narrower teeth than normal subjects espe-cially in posterior segments, which may have clinical implications during the orthodontic treatment process.

## Introduction

The Bolton analysis has been designed based on a constant proportion between the sum of the size of the mesiodistal dimension of maxillary to mandibular teeth, and is widely used as the most recognized method for diagnosing tooth size discrepancies. An ideal anterior ratio when the 6 anterior teeth in each arch are measured (anterior Bolton Index) is approximately 77.2% ± 1.65. The sum of the mesiodistal width of teeth in each arch from first molar to first molar (12 teeth; total Bolton index) should ideally be 91.3% ± 1.91. These values have been named as Bolton’s ratios. They are extremely helpful for diagnosis and treatment planning of orthodontic patients and can be used for determining the treatments’ functional and esthetic outcomes.^1,2^ Bolton in 1958 calculated that a constant proportion between the upper and the lower dentition should be present to achieve a perfect occlusion.^1,3^ Researchers have announced values higher than two times the mean and standard deviation values reported by Bolton as a significant discrepancy.^2,4^

Bolton’s ratios aid the orthodontists to gain some knowledge about the final post-treatment result without the need for diagnostic setups. Clinically, Bolton analysis has been used for determining the need for tooth size reduction through interproximal stripping or the addition of tooth size by composite resin restorations.^1,2^ Also, Bolton analysis can help orthodontists in treatment of patients with severe tooth size discrepancies.^5^ Nonetheless, it has some limitations and its precision and dependence to other factors are still matters of discussion.^6^ For instance, Bolton’s studied population and their ethnicity were not exactly specified; whereas, there is evidence regarding the presence of differences between various ethnicities in terms of tooth size discrepancies. In other words, differences in tooth size are not similar in all populations.^7^ Blacks have larger canines, premolars and first molars compared to whites, while there is no difference in size of maxillary central and lateral incisors between blacks and whites. Also, difference in tooth size of men and women is not similar for all teeth.^8^ Since ethnic and population-based differences in size of maxillary teeth do not always match those of mandibular teeth, different interarch relationships can be expected.

The present study aimed at assessing and comparing Bolton’s ratios before and after orthodontic treatment in skeletal class I patients presenting to a Department of Orthodontics.

## Materials and Methods

This retrospective descriptive-analytical study was conducted on 200 study casts of 100 patients treated in the Department of Orthodontics at Shahid Beheshti University of Medical Sciences. The samples were selected from 1,700 patient files present in the archives of this department. The inclusion criteria were as follows: having permanent dentition and having skeletal class- I occlusion.

Also, the exclusion criteria were as follows: hypoplasia or dental anomaly, tooth missing, history of previous orthodontic treatments before presenting to the university clinic, and extensive restorations, casting restorations or cusp coverage.

Demographic characteristics of patients including their first and last names, age, sex and file number were recorded. Other variables were including: approved treatment planning for tooth size discrepancy, pretreatment Bolton’s ratios based on the evidence present in patient files, measured sum of the mesiodistal widths of maxillary and mandibular teeth from the right first molar to the left first molar before and after treatment (by the research team), calculated values of the anterior and total Bolton indexes pre- and post-treatment.

Measurement of tooth dimensions: Measurement of tooth dimensions was done using a digital caliper with 0.1 mm accuracy. For all teeth, caliper branches were moved parallel to the longitudinal axis of teeth and the greatest mesiodistal width was recorded at the level of contact points. Measurements were done twice for each tooth with a time interval. If difference in measurements was more than 0.1 mm, another measurement would be done for the third time and the mean value would be used for statistical analysis. The obtained values were put in the relevant formula and the two Bolton’s ratios were calculated for each subject.^2^

Fisher’s exact test was used to evaluate the difference between male and female samples in terms of normal and abnormal range of anterior and total ratios. Pre- and post-treatment values for anterior and total indexes were evaluated using Paired t-test, and Pearson’s correlation coefficient was employed for assessing the difference between mesiodistal widths of the same tooth on both sides of the dental arch in each jaw. One sample t test was used for statistical comparison of the ratios obtained in this study with the ideal values reported by Bolton.

## Results

Pre-treatment ABI was abnormal in 95% of samples. This index was within the normal range only in 5% of samples. Pre-treatment TBI was abnormal in 97% of samples and normal in only 3%.

Post-treatment ABI was abnormal in 83% of patients and within the normal range in 17%. Post-treatment TBI was abnormal in 85% of subjects and normal in 15%.

Fisher’s exact test could not find a significant difference between males and females in terms of frequency of normal and abnormal ABIs before treatment (P = 0.12). The same results were found for TBIs (P = 0.615).

Evaluation of the compatibility and success of offered treatment plan for resolving tooth size discrepancies:

- Compatibility is defined as matching the treatment with the inter-maxillary tooth size discrepancy
- Successful treatment is defined as resolving the discrepancy and instating normal Bolton’s ratios

First Assessment was done among a group of patients (32 subjects) who had below normal post-treatment ABI values (Table 3).

**Table 3:** Frequency distribution of subjects with TBI less than normal, normal and above normal before and after the orthodontic treatment

After treatment / Before treatment	Less than normal	Normal	Above normal	Total
Less than normal	19%	4%	7%	30%
Normal	0%	3%	0%	3%
Above normal	29%	8%	30%	67%
Total	48%	15%	37%	100%

In 53% of these patients (17 subjects), the treatment plan was compatible with the tooth size discrepancy. In other words, if ABI was less than normal interproximal tooth size reduction was done for maxillary teeth or dental material was added to the mandibular teeth and vice versa. However, after treatment, ABI was still not within the normal range and thus, the treatment plan in this respect was not completely successful.

In 19% of these patients (6 subjects), changes in tooth size were made in the arch opposing the causative jaw (incompatible and unsuccessful treatment plan). In these patients, cephalometric analysis of angles related to maxillary and mandibular incisors revealed that in all of the mentioned subjects, incisal angles of the causative arch (1 to SN or IMPA)^9^ and subsequently the incisal angles of the opposing jaw increased after treatment compared to the pre-treatment values. In other words, patients’ crowding was resolved by protruding the teeth without the need for tooth size reduction.

In 22% of patients (7 subjects) tooth size alterations had been performed in both maxilla and mandible (incompatible and unsuccessful treatment plan). In 6% (2 subjects) pre-treatment ABI was normal. In this group, considering the need for retrusion of protruded mandibular incisors, stripping was done and this treatment decreased the ABI. Tooth material reduction in this group was 1 mm which is not significant.

The second assessment was done among a group of patients (51 subjects) with higher than normal ABI after treatment (Table 1).

**Table 1:** Frequency distribution of ABI values below, within or above the Bolton’s normal range before and after orthodontic treatment

After treatment/Before treatment	Below normal	Normal	Above normal	Total
Below normal	12%	0%	10%	22%
Normal	2%	2%	1%	5%
Above normal	18%	15%	40%	73%
Total	32%	17%	51%	100%

In 84% of these patients (43 subjects) treatment plan was compatible but not completely successful.

In 6% (3 subjects), changes in tooth size were made in the arch opposing causative jaw (incompatible and unsuccessful treatment plan).

In 8% of these patients (4 subjects) tooth size alterations had been performed in both arches (incompatible and unsuccessful treatment plan).

Totally, 2% (1 subject) had normal ABI before treatment. Based on the need to retrude their protruded maxillary incisors, stripping had been performed, which increased the ABI. Tooth size alterations were within 1 mm range which does not have clinical significance.

As seen in Table 2, in 77% of patients treatment plans were compatible with ABI discrepancies. The treatment plan was completely successful in 17% of subjects.

**Table 2:** Frequency distribution of treatment plans in terms of compatibility and success in correcting the ABI discrepancies

Treatment plan aiming at correcting the ABI in patients	Number of patients
Compatible and completely successful	15%
Compatible but not completely successful	60%
Incompatible and unsuccessful	23%
No change and within the normal range	2%

The third assessment was done among 48 patients who had TBI values lower than normal after the treatment (Table 3).

In 67% of these patients (32 subjects), the treatment plan was compatible but not completely successful.

In 23% (11 subjects), tooth size alterations were done in the arch opposing the causative arch (incompatible and unsuccessful treatment plan). In all these patients the angles of incisors in the arch with excessive tooth material had increased and by subsequent gaining of space, the patients’ crowding had been resolved.

In 10% of these patients (5 subjects), tooth size alterations had been performed in both arches (incompatible and unsuccessful treatment plan).

The fourth assessment was done among 37 patients who had post-treatment TBI above the normal limit (Table 3).

In 73% of these patients (27 subjects), the treatment plan was compatible but not completely successful.

In 13% (5 subjects), tooth size alterations had been done in the arch opposing the causative arch (treatment plan was incompatible and unsuccessful). In these patients, the angles of incisors had been increased in the arch causing the discrepancy which had resulted in subsequent space gain and obviated the need for tooth size reduction. In 14% of patients (5 subjects) tooth size alterations had been done in both arches, i.e. treatment plan was incompatible and unsuccessful.

As seen in Table 4, in 74% of patients the treatment plan was compatible with TBI discrepancies and in the right direction to correct them. Treatment was completely successful in 12% of these patients.

**Table 4:** Frequency distribution of treatment plans in terms of compatibility and success in correcting the TBI discrepancies

Treatment plan for correction of patients’ TBI discrepancy	Number of patients
Compatible and completely successful	12%
Compatible but not completely successful	59%
Incompatible and unsuccessful	26%
No change, within the normal range	3%

## Discussion

Bolton’s study which was the basis of further studies was conducted on 55 models with excellent occlusion, out of which 44 had undergone orthodontic treatment and 11 cases had not been treated.^1,10^ In our study, 100 skeletal class I occlusion patients who had undergone fixed orthodontic treatment were selected and evaluated. Therefore, a statistical comparison between our samples and those of Bolton’s study was feasible. Based on the present study results, after measurements and calculations of ABI and TBI of patients after treatment, the mean ABI value was 77.35% ± 2.14%. Therefore, the mean ABI value after treatment in our patients was closer to the value reported by Bolton and was only slightly higher. The reason can be different populations and ethnicities. The difference in ABI values between our study and that of Bolton was not statistically significant (P > 0.05).

In our study, the mean TBI of patients after treatment was 91.2% ± 1.66%. The mean TBI value in the present study was very close to the TBI value reported by Bolton. Our obtained mean TBI value was only slightly lower than that of Bolton. As mentioned for ABI, the small difference between our results and those of Bolton can be justified by the differences in the studied populations and ethnic groups. Also, the smaller difference between our obtained mean TBI value and that of Bolton compared to ABI may be due to the greater manipulations and alterations in tooth size in the anterior segment especially the inter-canine space.

After comparing our obtained standard deviations and range of ABI and TBI with those of Bolton, we noticed that our values had a higher dispersion. Similar results in this respect were reported by Salehi et al,^11^ Freeman et al,^12^ and Crosby and Alexander.^4^ By comparing the results of Freeman et al^12^ with those of the present study, we found no statistically significant difference in TBI or ABI between the 2 studies (P > 0.05). The values reported by Freeman et al^12^ were only slightly higher than those of ours. This difference can be attributed to the ethnic dimorphism in Freeman et al, study.^12^

Santaro et al reported the TBI to be 91.3% (similar to Bolton) among the Dominican Americans. Smith et al evaluated Bolton’s ratios in Black, White and Hispanic populations and reported the mean TBI values of 92.3%, 93.4%, and 93.1%, respectively. The ABI for these populations was 79.6%, 79.3% and 80.5%, respectively. These rates revealed significant differences when compared with those of Bolton and also with one another.^5^

Salehi et al^11^ evaluated the mean ABI as77.2% in Iranian patients in Shiraz. The TBI was estimated as 90.6%. No significant differences were detected between these values and those of Bolton. Freeman et al, reported the mean overall ratio and mean anterior ratio in their samples as 91.4% ± 2.57% and 77.8% ± 3.07%, respectively.^12^ Mirzakouchaki et al^13^ evaluated tooth size ratios in an Iranian-Azari population and reported the ABI and TBI values of 78.0% ± 3.1% and 92.0% ± 2.4%, respectively. Lopatiene and Dumbravaite^14^ reported the mean ABI and TBI values to be 77.89% ± 4.29% and 92.74% ± 2.49%, respectively. Jaiswal and Paudel^15^ evaluated a Nepalese population and reported these values to be 79.46% ± 2.6% and 92.42% ± 1.8%, respectively. Adeyemiet al^16^ assessed tooth size ratios of a Nigerian population and stated the mean TBI and ABI values to be 92.5% ± 0.5% and 79.0% ± 0.5%, respectively.

Different values reported for ABI and TBI in various studies can be due to ethnic differences,^5,8,17^ and extensive morphological variations of maxillary incisors among the studied populations.^12,18,19^ Araujo and Souki^20^ concluded that higher prevalence of tooth size discrepancies among their samples can be explained by the higher degree of genetic mixing in the Brazilian population. These results emphasized the need for development of more specific standards for different populations.

Kachoei et al^21^ studied Bolton’s ratios among 12-14 year-old Iranians and reported the mean ABI and TBI to be 78.1% ± 0.28 and 92.24% ± 0.21, respectively. These values were slightly higher than ours but this difference was not statistically significant. This slight difference seems to be due to the method of sampling in the latter study as they included age and sex limits in their inclusion criteria and only adolescents in the age range of 12-14 years and equal number of girls and boys were entered the study.^21^

In our study, no significant difference was detected between girls and boys in terms of frequency of normal and abnormal ABI and TBI values (before the treatment). Kachoei et al^21^ could not find significant differences between girls and boys in terms of ABI and TBI values. The present study findings indicated higher but not statistically significant Bolton’s ratios in males. On the other hand, it has been documented that the differences that exist in ABI and TBI between genders can also be related to ethnicity and different populations.^5,22,23^

In the clinical setting, treatment plans for tooth size discrepancies (Bolton’s) are based on the estimation of tooth material excess (width) in millimeter.

## Conclusion

Based on the results of the present study, the ratios calculated by Bolton are applicable among the studied population where performed orthodontic treatments have been generally successful in correcting the tooth size discrepancies.
